# Gut‐Liver Translocation of *Bacteroides Uniformis* Alleviates Advanced Metabolic Dysfunction‐Associated Steatotic Liver Disease by Suppressing Hepatocyte Ferroptosis via Propionic Acid Secretion

**DOI:** 10.1002/advs.76865

**Published:** 2026-07-27

**Authors:** Haiyang Liu, Zun Fan, Zhen Liu, Lei Qin, Xin Zhao

**Affiliations:** ^1^ Department of General Surgery the First Affiliated Hospital of Soochow University Suzhou China

**Keywords:** *Bacteroides uniformis*, gut‐liver axis, hepatocyte ferroptosis, liver‐resident microbiota, metabolic dysfunction‐associated steatotic liver disease, propionic acid

## Abstract

Interactions between hepatic metabolism and the gut microbiota play a crucial role in the pathogenesis of metabolic dysfunction‐associated steatotic liver disease (MASLD), highlighting the gut‐liver axis as a promising therapeutic target. However, little is known about how gut dysbiosis influences the composition of liver‐resident microbiota, and how this alteration contributes to disease progression. In this study, 16S rRNA sequencing of liver samples from patients with advanced MASLD reveals a marked reduction in the abundance of *Bacteroides uniformis* (*B. uniformis*), a trend also observed in fecal samples. These findings are replicated in a mouse model, where *B. uniformis* levels are decreased in both hepatic and gut microbial communities. Notably, gavage of *B. uniformis* increases its abundance in the liver and attenuates multiple features of advanced MASLD. Further investigation identifies propionic acid (PA) as a key mediator responsible for the protective effects of *B. uniformis*. Mechanistically, both *B. uniformis* and PA administration suppress lipid peroxidation and ferroptotic stress in hepatocytes through activating mammalian target of rapamycin phosphorylation and upregulating glutathione peroxidase 4 expression. Taken together, our results uncover a distinctive liver‐resident microbiota in advanced MASLD and underscore the therapeutic potential of promoting gut‐liver translocation of *B. uniformis* to curb disease progression.

## Graphical abstract

Systematic characterization of the liver‐resident microbiota in advanced metabolic dysfunction‐associated steatotic liver disease (MASLD) reveals a consistent depletion of *Bacteroides uniformis (B. uniformis)* in both hepatic and gut microbial communities. Oral gavage of *B. uniformis* promotes its translocation from the gut to the liver and reverses MASLD progression. Mechanistically, hepatic *B. uniformis* produces the metabolite propionic acid (PA), which suppresses hepatocyte ferroptosis.

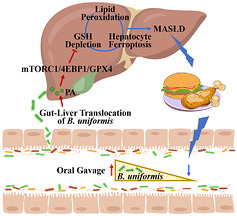

## 1 Introduction

Metabolic dysfunction‐associated steatotic liver disease (MASLD), a metabolic disorder characterized by excessive lipid accumulation in the liver, is a pressing health concern and affects approximately 25% of the adult population worldwide [1, 2]. This disease spectrum ranges from simple steatosis to metabolic dysfunction‐associated steatohepatitis, with progression to hepatic fibrosis, cirrhosis, and hepatocellular carcinoma (HCC) [3, 4]. In fact, MASLD has become a leading cause of end‐stage liver disease and the fastest‐growing indication for liver transplantation [5]. However, despite these alarming trends, there is still a lack of pharmacological treatments for advanced conditions [6]. The linkage between hepatocellular death and MASLD progression is well‐documented, and current studies reveal that steatotic hepatocytes are particularly susceptible to ferroptosis, an iron‐dependent cell death modality executed by lipid peroxidation [7, 8]. When lipid peroxides irreversibly damage cell membranes, ferroptotic hepatocytes adopt a secretory phenotype, releasing a cascade of damage‐associated molecular patterns and cytokines. These molecules not only recruit monocyte‐derived macrophages (Mo‐Macs) to perpetuate lobular inflammation, but also activate hepatic stellate cells (HSCs) to produce fibrous scar [9]. As such, researchers are exploring ferroptosis suppression as a potential treatment for advanced MASLD, using iron chelators (e.g., deferoxamine) or lipophilic antioxidants (e.g., ferrostatin‐1, liproxstatin‐1), as well as genetically boosting glutathione (GSH) production by targeting key regulators like glutathione peroxidase 4 (GPX4) and system Xc^−^ cystine/glutamate antiporter (a transmembrane protein complex containing SLC7A11/xCT subunit) [10, 11, 12]. While these anti‐ferroptosis therapies show promise in preclinical studies, their clinical transition is hindered by the risk of disrupting normal biological processes.

Recent advances in microecology have shed light on the therapeutic benefit of modulating host‐microbe interactions [13]. Growing evidence indicates that microbes and their metabolic products can reshape the hepatic microenvironment during chronic injury, impact the balance of cell proliferation and death, regulate the immune system and healing response, thereby either accelerating or alleviating MASLD progression [14, 15, 16]. To date, research on host‐microbe interactions predominantly centers on the gut microbiota. Clinical investigations find close correlations between gut dysbiosis and MASLD progression, while animal models provide causal evidence through gut microbial manipulation [17, 18, 19]. Strikingly, strategies including fecal microbiota transplantation, antibiotics, and probiotics have shown efficacy in restoring gut microbial homeostasis and improving MASLD outcomes.

As the first organ exposed to gut microbes via portal vein blood drainage, the liver was supposed to be sterile for years. Until recently, the development of next‐generation sequencing technology has revolutionized our understanding of liver‐resident microbiota [20]. Albeit at low biomass, these microbial communities are now recognized as an essential component of the hepatic microenvironment, and their association with liver diseases appears even stronger than that of the gut microbiota [21, 22]. Comparative studies have described a distinct composition of microbial community in intratumor liver tissues. For instance, Guo et al. reported elevated abundances of *Neisseria*, *Clostridia_UCG‐014*, *Fusobacterium*, and *Lactobacillus* in HCC, whereas *Dietzia*, *Faecalibacterium*, *Megamonas*, *Hydrogenophaga*, and *Agathobacter* were reduced [23]. Beyond compositional differences, functional studies have demonstrated that the rational use of liver‐resident microbes may offer novel therapeutic strategies. Cai et al. reported that *Paraburkholderia fungorum* supplementation protected mice from intrahepatic cholangiocarcinoma by inhibiting cancer cell migration and proliferation through amino acid metabolism [24]. Such findings underscore the pivotal role of liver‐resident microbiota in tumor development and treatment response. However, as we know, the hepatic microbial landscape in MASLD progression remains poorly characterized, warranting detailed exploration of how these microbial communities interact with steatotic hepatocytes.

In this study, we conducted 16S rRNA sequencing on liver samples obtained from clinical patients and mouse models with advanced MASLD. Our results revealed a strong correlation between hepatic microbial dysbiosis and disease progression. A notable reduction in the abundance of *Bacteroides uniformis* (*B. uniformis*) was observed in both the liver and gut of individuals with advanced MASLD, indicating its potential probiotic function. Moreover, intragastric administration of *B. uniformis* in mice increased its hepatic abundance and led to a marked resolution of hepatic steatosis, hepatocyte ballooning, lobular inflammation, HSC activation, and perisinusoidal fibrosis. We also identified propionic acid (PA), a metabolite produced by *B. uniformis*, as a key mediator alleviating advanced MASLD symptoms. Mechanistically, live *B. uniformis* could support hepatocyte survival by PA‐mediated suppression of lipid peroxidation and ferroptotic stress through activating mammalian target of rapamycin (mTOR) phosphorylation. Overall, our findings reveal a critical interaction between the liver‐resident microbiota and steatotic hepatocytes, and establish the restoration of hepatic *B. uniformis* via gut‐liver translocation as a promising therapeutic strategy for advanced MASLD.

## 2 Results

### 2.1 Alterations in the Liver‐Resident Microbiota of Patients With Advanced MASLD

**FIGURE 1:**
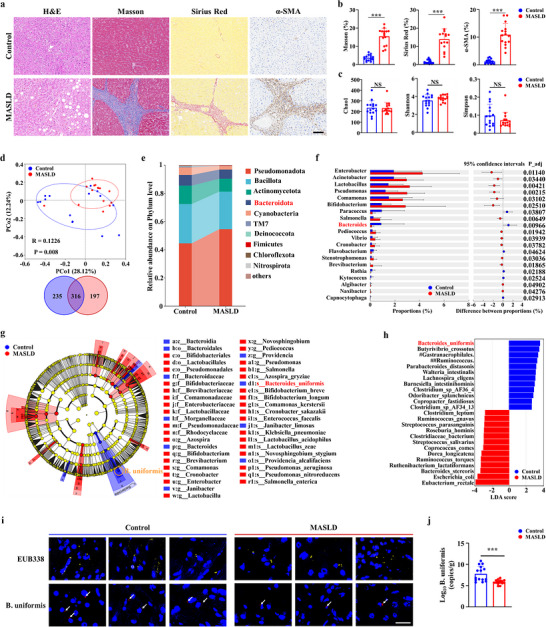
Alterations in the liver‐resident microbiota of patients with advanced MASLD. (a) Representative images of human liver samples after staining with H&E, Masson, Sirius Red, and α‐SMA antibody. Scale bar = 100 µm. (b) Positive areas of Masson, Sirius Red, and α‐SMA staining between the control and MASLD groups (*n* = 15). (c) Comparison of hepatic microbial richness (α‐diversity) between the two groups based on the Chao1, Shannon, and Simpson indices (*n* = 15). (d) Comparison of hepatic microbial composition (β‐diversity) between the two groups based on the PCoA (*n* = 15). (e) Stacked bar plot showing the relative abundance of hepatic microbial communities at the phylum level between the two groups (*n* = 15). (f) Wilcoxon rank‐sum test with Bonferroni correction identifying hepatic microbial genera with differential abundance between the two groups (*n* = 15). (g) LEfSe‐derived cladogram identifying differentially abundant taxa across all taxonomic levels between the two groups (*n* = 15). (h) LEfSe analysis identifying differentially abundant microbial species in human fecal samples between the two groups (*n* = 15). (i) FISH images showing the presence of bacteria (yellow) and *B. uniformis* (red) in human liver samples from the two groups. Scale bar = 20 µm. (j) Relative abundance of *B. uniformis* in human liver samples detected by qPCR (n  =  15). The data were presented as the mean ± SD. Statistical differences were analyzed using an unpaired two‐tailed Student's t‐test. ^***^ *p* < 0.001, NS: not significant. #Gastranaerophilales.: Candidatus_Gastranaerophilales_bacterium, ##Ruminococcus.: Ruminococcus_sp_BSD2780120874_150323_B10.

To decipher the relationship between liver‐resident microbiota and MASLD progression, we enrolled 30 participants undergoing liver resection, whose clinical characteristics were summarized in Table S1. Among them, 15 participants were diagnosed with advanced MASLD (Figure 1a,b), as evidenced by hepatocyte ballooning, lobular inflammation (H&E staining), HSC activation (α‐SMA staining), and perisinusoidal fibrosis (Masson and Sirius Red staining). We subsequently conducted 16S rRNA sequencing on liver samples. Following stringent quality control and removal of background contaminants, a total of 30 phyla, 54 classes, 132 orders, 353 families, 748 genera, and 2360 species were identified. Although α‐diversity analysis (Chao1, Shannon, and Simpson indices) showed no significant differences in microbial richness between the control and MASLD groups (Figure 1c), principal coordinate analysis (PCoA) revealed clear separation (Figure 1d), suggesting a compositional discrimination in the liver‐resident microbiota. Venn diagram analysis identified 235 and 197 genus‐level microbes unique to the control and MASLD groups, respectively, with 316 shared between the two (Figure 1d). Together, these results indicated that MASLD progression was associated with hepatic microbial dysbiosis in clinical patients.

At the phylum level, both groups showed predominant enrichment of *Pseudomonadota*, *Bacillota*, *Actinomycetota*, *Bacteroidota*, and *Cyanobacteria* (Figure 1e). At the genus level, the MASLD group exhibited a significantly higher abundance of *Enterobacter*, *Acinetobacter*, *Lactobacillus*, *Pseudomonas*, and *Comamonas* compared to the control group, whereas the abundance of *Paracoccus* and *Bacteroides* was markedly reduced (Figure 1f). To comprehensively compare the hepatic microbial communities between the two groups, we performed a high‐dimensional taxonomic analysis using linear discriminant analysis (LDA) with effect size (LEfSe), which identified 44 differentially abundant taxa across all taxonomic levels (from phylum to species; LDA > 3, *p* < 0.05; Figure 1g and Figure S1). Notably, *B. uniformis*, a short‐chain fatty acid (SCFA)‐producing species belonging to the genus *Bacteroides*, showed an inverse correlation with MASLD progression. Fluorescence in situ hybridization (FISH) and absolute quantification via quantitative polymerase chain reaction (qPCR) further confirmed the presence of *B. uniformis* in the livers of both groups, with a significant reduction in its abundance in the MASLD group (Figure 1i,j). Additionally, we analyzed gut microbial composition using publicly available metagenomic datasets (NCBI SRA/ENA accessions PRJNA373901 and PRJEB6070), comprising fecal samples from 15 patients with advanced MASLD and 15 healthy controls (Table S2). As shown in Figure 1h and Figure S2, gut *B. uniformis* exhibited the most significant depletion in the MASLD group. Collectively, these findings indicated that a loss of *B. uniformis* in the gut might drive a parallel decline in the liver, which appeared to play an important role in the pathogenesis of advanced MASLD.

### 2.2 Validation of Decreased Hepatic *B. Uniformis* in Mice With Advanced MASLD

**FIGURE 2:**
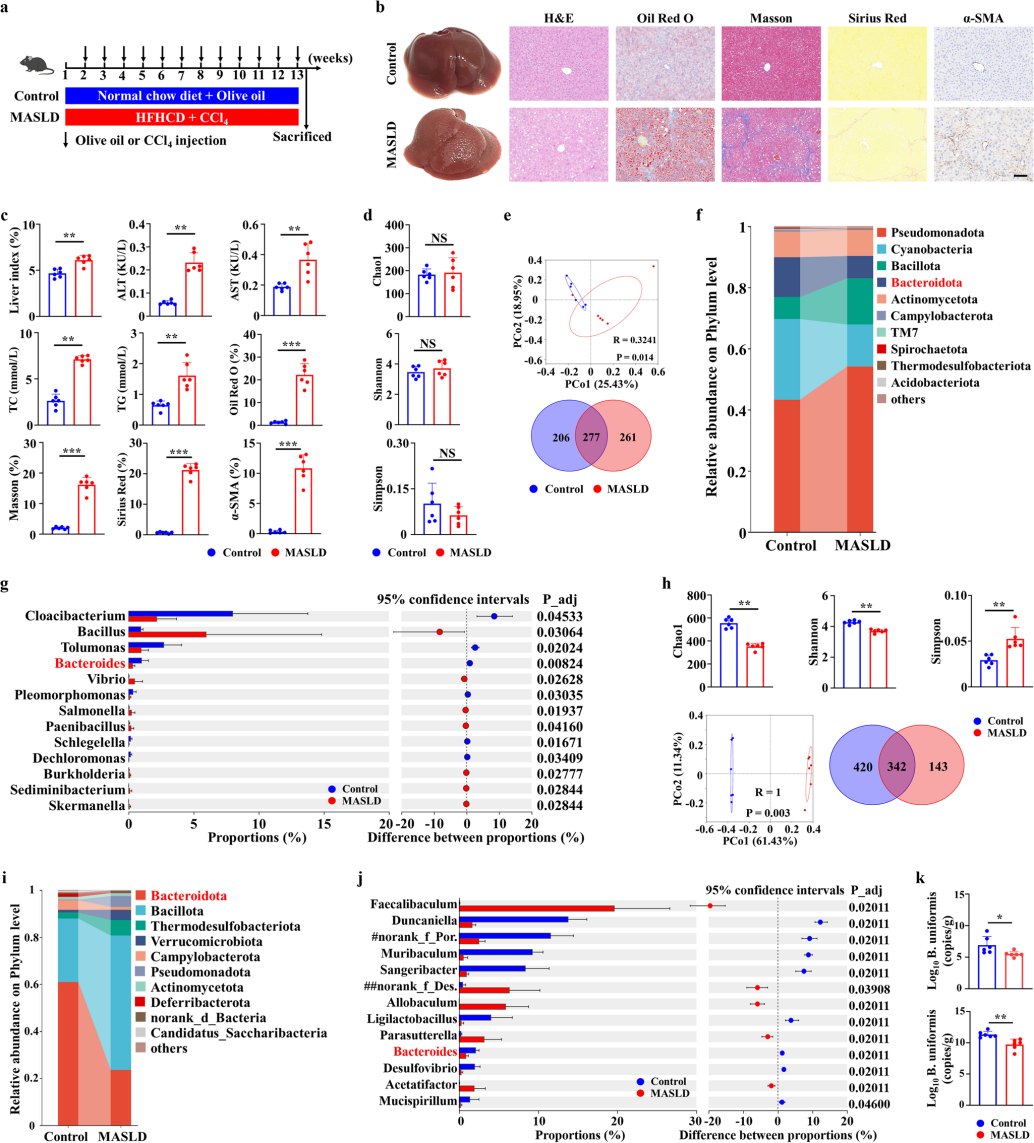
Validation of decreased hepatic *B. uniformis* in mice with advanced MASLD. (a) Schematic of experimental design, with black arrows indicating intraperitoneal injections of olive oil or CCl_4_ (*n* = 6). (b) Representative images of mouse liver samples after staining with H&E, Oil Red O, Masson, Sirius Red, and α‐SMA antibody. Scale bar = 100 µm. (c) Quantitative analysis of liver index, serum ALT, AST, TC, and TG levels, as well as positive areas of Oil Red O, Masson, Sirius Red, and α‐SMA staining between the control and MASLD groups (*n* = 6). (d) Comparison of hepatic microbial richness (α‐diversity) between the two groups based on the Chao1, Shannon, and Simpson indices (*n* = 6). (e) Comparison of hepatic microbial composition (β‐diversity) between the two groups based on the PCoA (*n* = 6). (f) Stacked bar plot showing the relative abundance of hepatic microbial communities at the phylum level between the two groups (*n* = 6). (g) Wilcoxon rank‐sum test with Bonferroni correction identifying hepatic microbial genera with differential abundance between the two groups (*n* = 6). (h) Comparison of gut microbial richness (α‐diversity) and composition (β‐diversity) between the two groups (*n* = 6). (i) Stacked bar plot showing the relative abundance of gut microbial communities at the phylum level between the two groups (*n* = 6). (j) Wilcoxon rank‐sum test with Bonferroni correction identifying gut microbial genera with differential abundance between the two groups (*n* = 6). (k) Relative abundance of *B. uniformis* in mouse liver (up) and fecal (down) samples detected by qPCR. The data were presented as the mean ± SD. Statistical differences were analyzed using an unpaired two‐tailed Student's t‐test. ^*^ *p* < 0.05, ^**^ *p* < 0.01, ^***^ *p* < 0.001, NS: not significant. #norank_f_Por.: norank_f_Porphyromonadaceae, ##norank_f_Des.: norank_f_Desulfovibrionaceae.

We next sought to validate the above findings in a mouse model of MASLD with rapid progression of extensive fibrosis. To establish this model, mice were fed a high‐fat high‐cholesterol diet (HFHCD) and received weekly intraperitoneal injections of carbon tetrachloride (CCl_4_) for 12 weeks. Control mice were fed a normal chow diet and received weekly injections of olive oil (Figure 2a). As expected, mice in the MASLD group showed increased liver‐to‐body weight ratio (liver index), along with elevated serum levels of alanine aminotransferase (ALT), aspartate aminotransferase (AST), total cholesterol (TC), and triglycerides (TG) compared to the control group (Figure 2c). Histological examination revealed hepatic steatosis, hepatocyte ballooning, lobular inflammation, HSC activation, and perisinusoidal fibrosis, confirming the development of advanced MASLD in HFHCD/CCl_4_‐challenged mice (Figure 2b,c).

To further explore the relationship between liver‐resident microbiota and MASLD progression, we performed 16S rRNA sequencing on liver samples from both groups of mice. Consistent with observations in clinical patients, HFHCD/CCl_4_ challenge induced significant shifts in the hepatic microbial composition of mice (Figure 2e). No differences, however, were observed in α‐diversity analysis between the two groups (Figure 2d). A Venn diagram illustrated 206 and 261 genus‐level microbes unique to the control and MASLD groups, respectively, with 277 shared between them (Figure 2e). Bar plot analysis at the phylum level showed that both groups were predominantly enriched in *Pseudomonadota*, *Cyanobacteria*, *Bacillota*, *Bacteroidota*, and *Actinomycetota*, a profile closely mirroring that seen in clinical samples (Figure 2f). At the genus level, the control group showed higher abundances of *Cloacibacterium*, *Tolumonas*, and *Bacteroides*, while the MASLD group was enriched in *Bacillus*, *Vibrio*, and *Salmonella* (Figure 2g). Notably, the genus *Bacteroides* exhibited a consistent decrease in both human and mouse livers with advanced MASLD.

We next compared gut microbial communities between the control and MASLD groups. A significant difference in α‐diversity analysis, as reflected by Chao1, Shannon, and Simpson indices, was observed, along with high intergroup variability in PCoA, reflecting substantial shifts in both richness and composition of the gut microbiota (Figure 2h). At the phylum level, *Bacteroidota* was the top bacterium enriched in both groups, followed by *Bacillota*, *Thermodesulfobacteriota*, and *Verrucomicrobiota* (Figure 2i). At the genus level, the abundance of gut *Bacteroides* was significantly reduced in the MASLD group compared to the control group, mirroring the trend observed in liver tissues (Figure 2j). Since 16S rRNA sequencing lacks sufficient resolution for species‐level identification, we performed absolute quantification using qPCR on both liver and fecal samples. Compared to their healthy counterparts, mice with advanced MASLD showed decreased levels of *B. uniformis* in the liver, a pattern that was also consistent in the gut (Figure 2k). Together, these findings indicated that MASLD progression in mice also led to a loss of *B. uniformis* in the gut, which in turn reduced its translocation to the liver and limited the enrichment of hepatic *B. uniformis*.

### 2.3 Gut‐Liver Translocation of *B. Uniformis* Alleviated MASLD Progression in Mice

**FIGURE 3:**
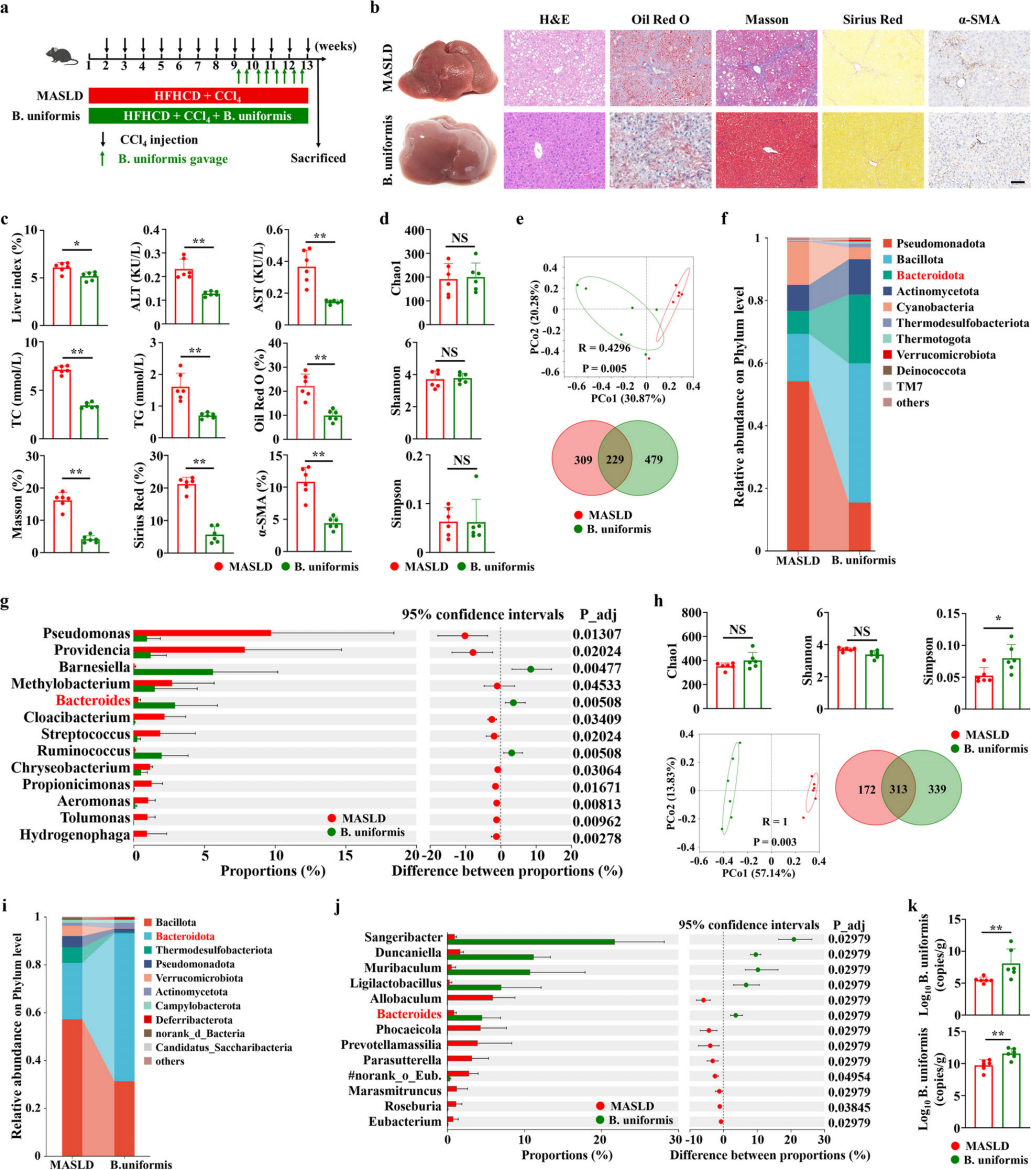
Gut‐liver translocation of *B. uniformis* alleviated MASLD progression in mice. (a) Schematic of experimental design, with green arrows indicating oral gavage of *B. uniformis* (*n* = 6). (b) Representative images of mouse liver samples after staining with H&E, Oil Red O, Masson, Sirius Red, and α‐SMA antibody. Scale bar = 100 µm. (c) Quantitative analysis of liver index, serum ALT, AST, TC, and TG levels, as well as positive areas of Oil Red O, Masson, Sirius Red, and α‐SMA staining between the MASLD and *B. uniformis* groups (*n* = 6). (d) Comparison of hepatic microbial richness (α‐diversity) between the two groups based on the Chao1, Shannon, and Simpson indices (*n* = 6). (e) Comparison of hepatic microbial composition (β‐diversity) between the two groups based on the PCoA (*n* = 6). (f) Stacked bar plot showing the relative abundance of hepatic microbial communities at the phylum level between the two groups (*n* = 6). (g) Wilcoxon rank‐sum test with Bonferroni correction identifying hepatic microbial genera with differential abundance between the two groups (*n* = 6). (h) Comparison of gut microbial richness (α‐diversity) and composition (β‐diversity) between the two groups (*n* = 6). (i) Stacked bar plot showing the relative abundance of gut microbial communities at the phylum level between the two groups (*n* = 6). (j) Wilcoxon rank‐sum test with Bonferroni correction identifying gut microbial genera with differential abundance between the two groups (*n* = 6). (k) Relative abundance of *B. uniformis* in mouse liver (up) and fecal (down) samples detected by qPCR. The data were presented as the mean ± SD. Statistical differences were analyzed using an unpaired two‐tailed Student's t‐test. ^*^ *p* < 0.05, ^**^ *p* < 0.01, NS: not significant. #norank_o_Eub.: norank_o_Eubacteriales.

Given the observed reduction of *B. uniformis* in both liver and fecal samples during MASLD progression, we administered *B. uniformis* by oral gavage to HFHCD/CCl_4_‐challenged mice to restore hepatic levels and evaluate its therapeutic potential (Figure 3a). Notably, *B. uniformis* administration slowed down the increase in liver index compared to the MASLD group (Figure 3c). Restoration of *B. uniformis* also reduced the serum levels of liver injury markers (ALT and AST) and lipid disorder markers (TC and TG). Subsequent histological examination of liver samples further corroborated these findings (Figure 3b). H&E and Oil Red O staining demonstrated that *B. uniformis* administration mitigated hepatic steatosis, hepatocyte ballooning, and lobular inflammation. Masson and Sirius Red staining showed a reduction in perisinusoidal fibrosis following *B. uniformis* administration. Additionally, immunohistochemical staining for α‐SMA observed a decreased proportion of activated HSCs in the liver after *B. uniformis* administration. Collectively, these findings indicated that gavage of *B. uniformis* markedly alleviated MASLD progression in mice.

16S rRNA sequencing was performed to investigate the effects of *B. uniformis* gavage on liver‐resident and gut microbiota. For liver‐resident microbiota, PCoA demonstrated that microbial composition in the *B. uniformis* group clearly separated from that in the MASLD group (Figure 3e). However, α‐diversity analysis revealed no significant differences between the two groups (Figure 3d). At the phylum level, *B. uniformis* administration led to a notable increase in the abundances of *Bacteroidota* and *Bacillota*, while the abundances of *Pseudomonadota* and *Cyanobacteria* were significantly reduced (Figure 3f). At the genus level, the abundances of *Bacteroides*, *Barnesiella*, and *Ruminococcus* were higher in the *B. uniformis* group than in the MASLD group (Figure 3g). For gut microbiota, the results from α‐diversity analysis and PCoA suggested that the microbial richness and composition were significantly changed after *B. uniformis* administration (Figure 3h). We also found that *B. uniformis* administration increased the abundances of both phylum‐level *Bacteroidota* and genus‐level *Bacteroides* (Figure 3i,j). Additionally, absolute quantification using qPCR on both liver and fecal samples observed an elevated level of *B. uniformis* following its gavage (Figure 3k). Together, these findings indicated that gavage of *B. uniformis* markedly increased its abundance in the gut, which might translocate to the liver and exert protective effects against MASLD progression.

### 2.4 Live *B. Uniformis* Alleviated MASLD Progression in Mice via PA Secretion

To investigate whether the probiotic function of *B. uniformis* against MASLD progression depended on bacterial viability, we administered pasteurized (non‐viable) *B. uniformis* by oral gavage to HFHCD/CCl_4_‐challenged mice (Figure S3). The results showed that non‐viable *B. uniformis* failed to rescue the elevation in liver index, liver injury markers (ALT and AST), or lipid disorder markers (TC and TG). Likewise, it did not promote the resolution of hepatic steatosis, hepatocyte ballooning, lobular inflammation, HSC activation, or perisinusoidal fibrosis. These findings indicated that live *B. uniformis* alleviated MASLD progression, possibly through active compound(s) secreted by this bacterium, rather than its abundance in the liver.

**FIGURE 4:**
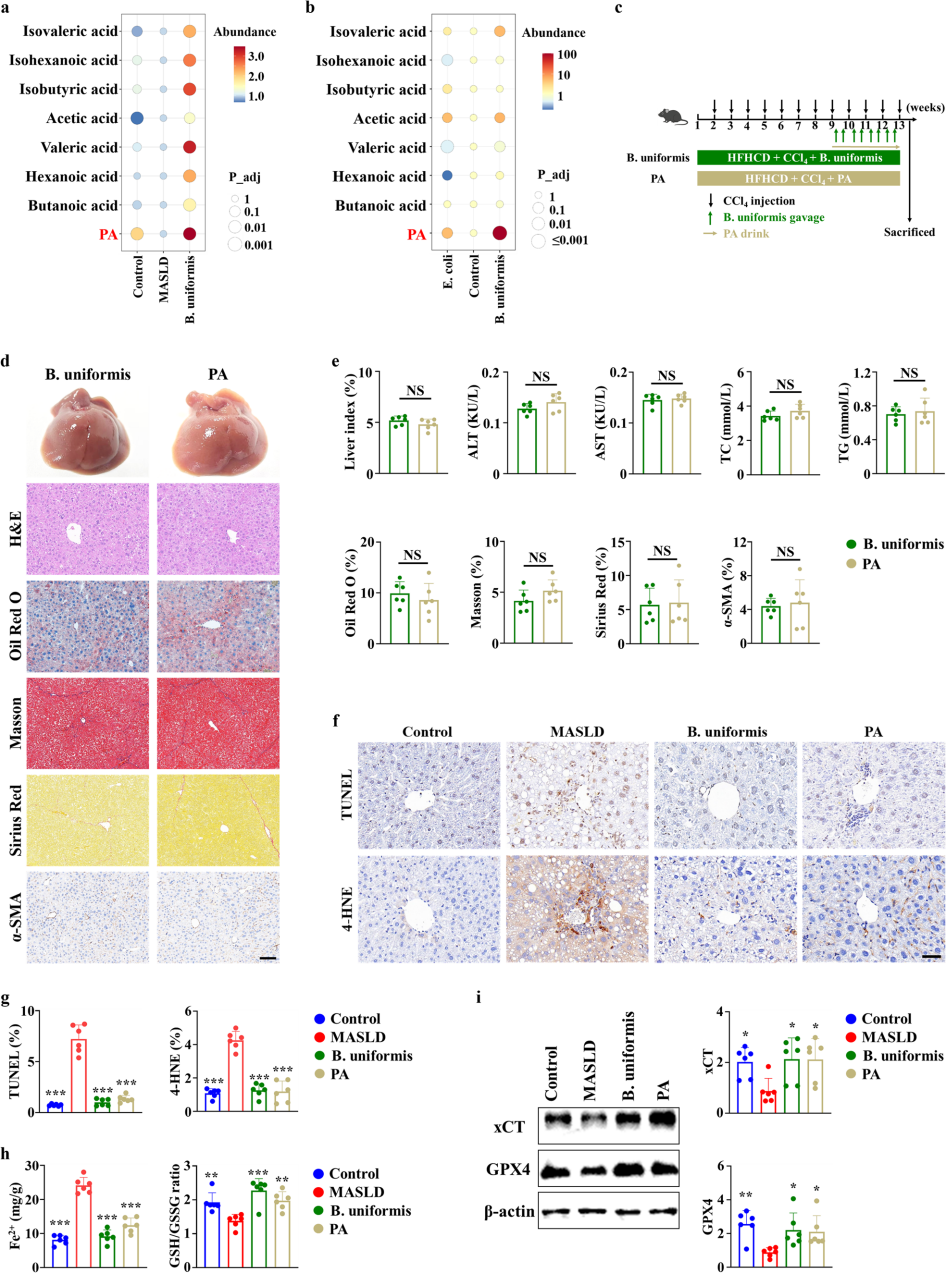
Live *B. uniformis* alleviated MASLD progression in mice via PA secretion. (a) Bubble plot highlighting the differentially expressed SCFAs in mouse liver samples from the control, MASLD, and *B. uniformis* groups (*n* = 6). (b) Bubble plot highlighting the differentially expressed SCFAs in culture medium from the blank control, *E. coli*, and *B. uniformis* (*n* = 3). (c) Schematic of experimental design, with a tawny arrow indicating PA supplementation in drinking water (*n* = 6). (d) Representative images of mouse liver samples after staining with H&E, Oil Red O, Masson, Sirius Red, and α‐SMA antibody. Scale bar = 100 µm. (e) Quantitative analysis of liver index, serum ALT, AST, TC, and TG levels, as well as positive areas of Oil Red O, Masson, Sirius Red, and α‐SMA staining between the *B. uniformis* and PA groups (*n* = 6). (f) Representative images of TUNEL and 4‐HNE staining in mouse liver samples from the control, MASLD, *B. uniformis*, and PA groups. Scale bar = 25 µm. (g) Quantitative analysis of TUNEL and 4‐HNE positive areas (*n* = 6). (h) Quantitative analysis of Fe^2+^ accumulation and GSH/GSSG ratio in mouse liver samples from the four groups (*n* = 6). (i) WB analysis of xCT and GPX4 in mouse liver samples from the four groups and corresponding quantifications (*n* = 6). The data were presented as the mean ± SD. Statistical differences were analyzed using an unpaired two‐tailed Student's t‐test or one‐way ANOVA followed by Tukey's post hoc test. ^*^ *p* < 0.05, ^**^ *p* < 0.01, ^***^ *p* < 0.001, NS: not significant.

Previous studies have reported that *B. uniformis* ferments carbon sources to produce SCFAs, which serve as energy substrates for both the bacterium and host [25, 26]. This process supports the maintenance of intraepithelial lymphocytes and type 3 innate lymphoid cells in the intestinal epithelium, thereby enhancing the first line of immune defense against dietary insults [27]. To determine whether SCFAs contributed to the therapeutic benefits of hepatic *B. uniformis*, we measured SCFA levels in mouse liver samples from the control, MASLD, and *B. uniformis* groups using liquid chromatography‐tandem mass spectrometry (LC‐MS/MS; Figure 4a). Compared to the control group, the MASLD group showed a significant reduction in PA. In contrast, acetic acid and isovaleric acid increased, while butanoic acid, hexanoic acid, valeric acid, isobutyric acid, and isohexanoic acid remained unchanged. Notably, *B. uniformis* administration restored PA production in the liver. We further confirmed the direct secretion of PA by *B. uniformis* in vitro (Figure 4b). After 48 h of incubation, the concentration of PA was significantly higher in the *B. uniformis* culture medium compared to both the blank control and the *Escherichia coli* (*E. coli*) culture medium. It was noteworthy that pasteurized *B. uniformis* failed to produce any SCFAs, including PA (Figure S4).

To investigate the role of PA in MASLD progression, we supplemented the drinking water of HFHCD/CCl_4_‐challenged mice with 200 mm PA for 4 weeks (Figure 4c). Specifically, this dosing regimen was established based on previous literature [28, 29], and in vitro findings from THLE‐2 cell experiments (Figure S5). No significant differences were observed between the *B. uniformis* and PA groups in terms of liver index, liver injury markers (ALT and AST), or lipid disorder markers (TC and TG; Figure 4e). Additionally, PA administration promoted the resolution of hepatic steatosis, hepatocyte ballooning, lobular inflammation, HSC activation, and perisinusoidal fibrosis, comparable to those seen with *B. uniformis* administration (Figure 4d). Collectively, these findings indicated that PA served as a key mediator through which hepatic *B. uniformis* alleviated the progression of MASLD.

### 2.5 *B. Uniformis* and PA Exerted Their Therapeutic Effects Through Suppression of Hepatocyte Ferroptosis

Emerging evidence links MASLD progression with hepatocyte ferroptosis, a form of metabolism‐dependent cell death driven by iron‐catalyzed lethal peroxidation of membrane lipids [7, 8, 9]. Although other forms of cell death are also upregulated in MASLD‐affected livers, ferroptosis is recognized as the principal mechanism. Moreover, recent studies have reported that PA can regulate iron homeostasis and protect epithelial cells from ferroptosis, thereby ameliorating symptoms of ulcerative colitis [30]. This prompted us to investigate whether suppression of hepatocyte ferroptosis might contribute to the therapeutic benefits of *B. uniformis* and PA in MASLD progression. Compared to the control group, the MASLD group exhibited more apoptotic cells (TUNEL staining), increased Fe^2+^ accumulation, elevated lipid peroxidation (4‐HNE staining), and a reduced GSH/GSSG ratio (Figure 4f–h). These results are consistent with earlier reports highlighting ferroptotic stress as a key driver of MASLD progression [10, 11, 12]. Importantly, both *B. uniformis* and PA administration greatly attenuated these ferroptosis‐related changes. Moreover, the expression of xCT and GPX4, two critical inhibitors of ferroptosis, was significantly downregulated in the MASLD group, but restored following administration of either *B. uniformis* or PA (Figure 4i). In line with existing literature, ferroptotic hepatocytes in advanced MASLD released high levels of inflammatory cytokines (TNF‐α, IL‐6, TGF‐β) and induced the infiltration of Mo‐Macs (Figure S6). In contrast, these inflammatory alterations were also reduced by *B. uniformis* and PA. Together, these findings indicated that suppression of hepatocyte ferroptosis might be a central mechanism through which *B. uniformis* and its metabolite PA conferred protection against MASLD progression.

**FIGURE 5:**
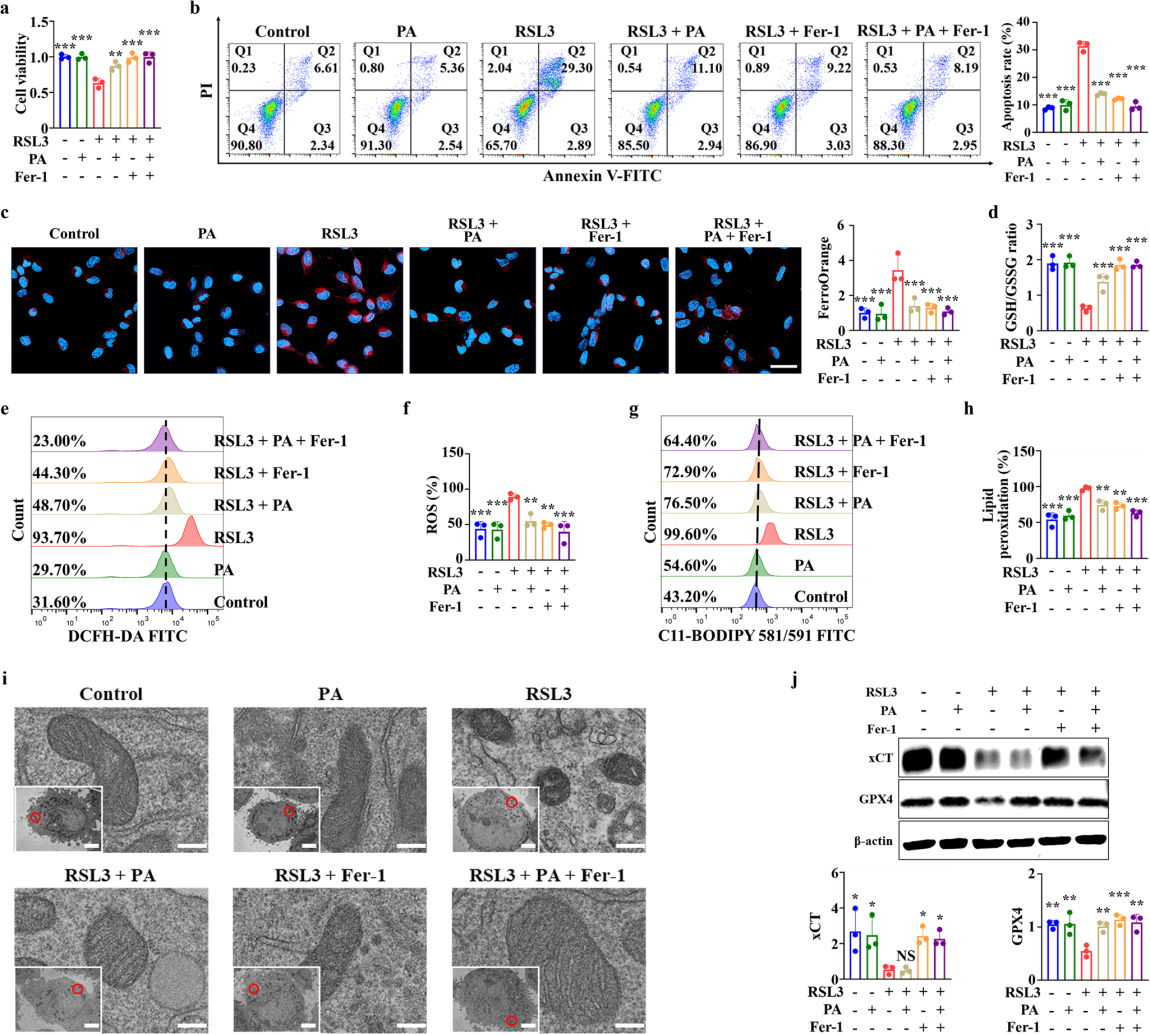
PA exerted its therapeutic effects through suppression of hepatocyte ferroptosis. (a) CCK‐8 assay to evaluate the cell viability in THLE‐2 hepatocytes treated with PBS, PA, RSL3, RSL3 + PA, RSL3 + Fer‐1, or RSL3 + PA + Fer‐1 (*n* = 3). (b) Annexin V/PI staining to evaluate the cell apoptosis in THLE‐2 hepatocytes subjected to different treatments (*n* = 3). (c) FerroOrange fluorescence to evaluate the Fe^2+^ accumulation in THLE‐2 hepatocytes subjected to different treatments (*n* = 3). Scale bar = 20 µm. (d) Quantitative analysis of the GSH/GSSG ratio in THLE‐2 hepatocytes subjected to different treatments (*n* = 3). (e) DCFH‐DA staining to evaluate the ROS levels in THLE‐2 hepatocytes subjected to different treatments. (f) Quantitative analysis of the ROS levels (*n* = 3). (g) C11‐BODIPY staining to evaluate the lipid peroxidation in THLE‐2 hepatocytes subjected to different treatments. (h) Quantitative analysis of the lipid peroxidation (*n* = 3). (i) TEM to detect the ultrastructure of mitochondria in THLE‐2 hepatocytes subjected to different treatments. Scale bars = 5 µm (inner) and 500 nm (outer). (j) WB analysis of xCT and GPX4 in THLE‐2 hepatocytes subjected to different treatments and corresponding quantifications (*n* = 3). The data were presented as the mean ± SD. Statistical differences were analyzed using a one‐way ANOVA followed by Tukey's post hoc test. ^*^ *p* < 0.05, ^**^ *p* < 0.01, ^***^ *p* < 0.001, NS: not significant.

We next evaluated the protective role of PA against RSL3‐induced ferroptosis in hepatocytes. RSL3 is a typical inducer of ferroptosis by targeting GPX4. Using the Cell Counting Kit‐8 (CCK‐8) assay and annexin V/propidium iodide (PI) staining, we observed that RSL3 exposure led to significant death in THLE‐2 cells (Figure 5a,b). This effect was suppressed by the specific ferroptosis inhibitor ferrostatin‐1 (Fer‐1), confirming that the observed cell death was triggered by ferroptotic stress. Notably, PA supplementation alleviated RSL3‐induced ferroptosis in a dose‐dependent manner, with maximum protection achieved at 5 mm (Figure S5). FerroOrange staining revealed that RSL3 exposure increased intracellular Fe^2+^ levels, an effect that was significantly reversed by both PA and Fer‐1 (Figure 5c and Figure S7). Further experiments observed elevated ROS levels (DCFH‐DA staining), enhanced lipid peroxidation (C11‐BODIPY staining), a reduced GSH/GSSG ratio, and mitochondrial condensation in RSL3‐induced THLE‐2 hepatocytes (Figure 5d–i). Similar to Fer‐1, PA supplementation restored these abnormalities. Additionally, PA supplementation prevented RSL3‐induced GPX4 degradation, though it did not protect against the loss of xCT (Figure 5j). The lack of an effect on xCT in vitro, in contrast to its restoration in vivo, likely reflects the complexity of the in vivo microenvironment, including paracrine signaling, extracellular matrix interactions, and metabolic gradients, which simplified in vitro systems cannot recapitulate. Collectively, these findings indicated that PA could mimic the action of ferroptosis inhibitor Fer‐1 and support hepatocyte survival under ferroptotic stress.

### 2.6 PA Suppressed Hepatocyte Ferroptosis by Targeting mTOR

**FIGURE 6:**
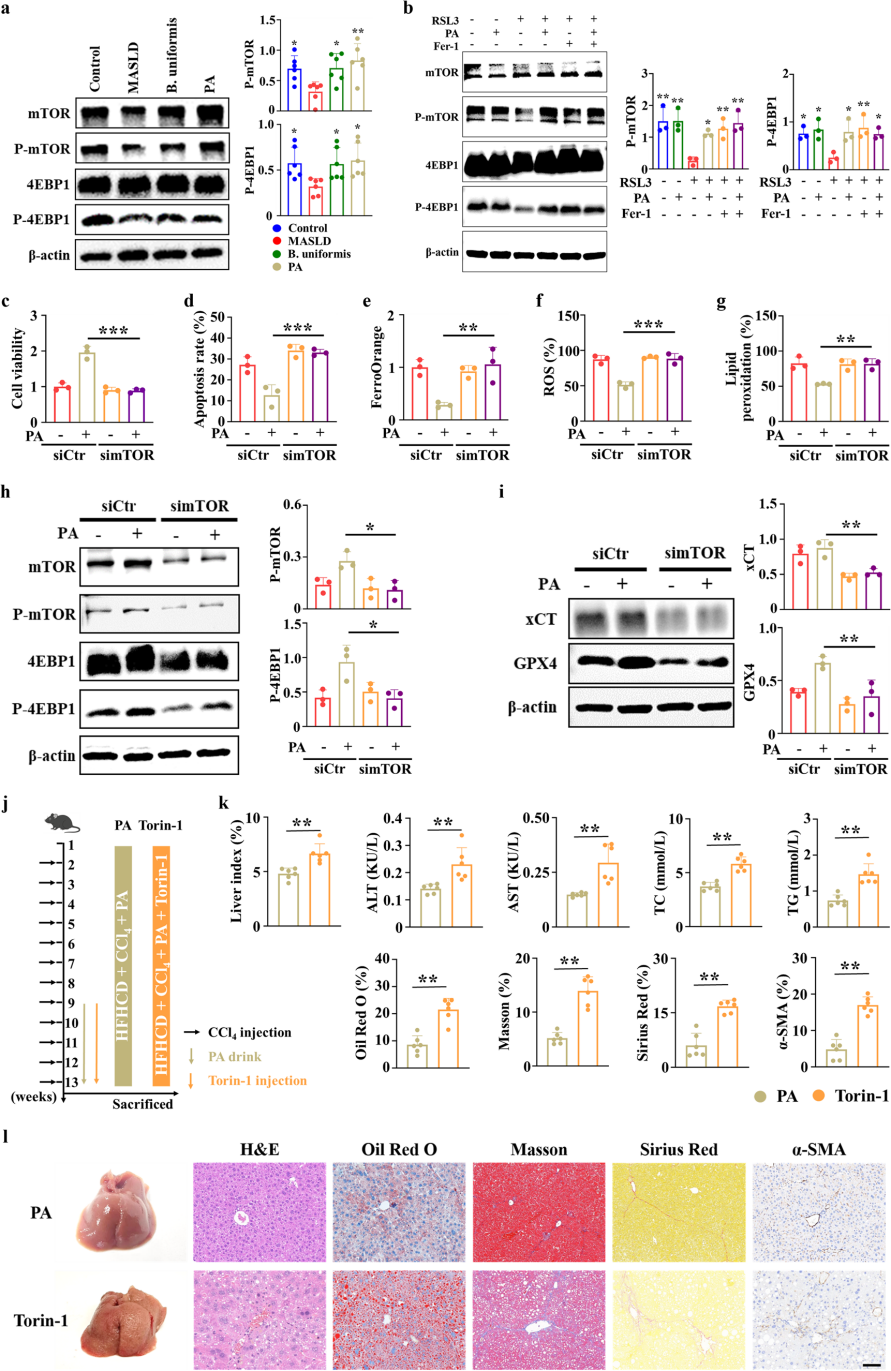
PA suppressed hepatocyte ferroptosis by targeting mTOR. (a) WB analysis of mTOR, p‑mTOR, 4EBP1, and p‑4EBP1 in mouse liver samples from the control, MASLD, *B. uniformis*, and PA groups, and corresponding quantifications (*n* = 6). (b) WB analysis of mTOR, p‑mTOR, 4EBP1, and p‑4EBP1 in THLE‐2 hepatocytes treated with PBS, PA, RSL3, RSL3 + PA, RSL3 + Fer‐1, or RSL3 + PA + Fer‐1, and corresponding quantifications (*n* = 3). (c) CCK‐8 assay to evaluate the cell viability in RSL3‐induced THLE‐2 hepatocytes treated with siCtr, PA + siCtr, simTOR, or PA + simTOR (*n* = 3). (d) Annexin V/PI staining to evaluate cell apoptosis in RSL3‐induced THLE‐2 hepatocytes subjected to different treatments (*n* = 3). (e) FerroOrange fluorescence to evaluate the Fe^2+^ accumulation in RSL3‐induced THLE‐2 hepatocytes subjected to different treatments (*n* = 3). (f) DCFH‐DA staining to evaluate the ROS levels in RSL3‐induced THLE‐2 hepatocytes subjected to different treatments (*n* = 3). (g) C11‐BODIPY staining to evaluate the lipid peroxidation in RSL3‐induced THLE‐2 hepatocytes subjected to different treatments (*n* = 3). (h) WB analysis of mTOR, p‑mTOR, 4EBP1, and p‑4EBP1 in RSL3‐induced THLE‐2 hepatocytes subjected to different treatments (*n* = 3). (i) WB analysis of xCT and GPX4 in RSL3‐induced THLE‐2 hepatocytes subjected to different treatments (*n* = 3). (j) Schematic of experimental design, with an orange arrow indicating intraperitoneal injections of Torin‐1 (*n* = 6). (k) Quantitative analysis of liver index, serum ALT, AST, TC, and TG levels, as well as positive areas of Oil Red O, Masson, Sirius Red, and α‐SMA staining between the PA and Torin‐1 groups (*n* = 6). (l) Representative images of mouse liver samples after staining with H&E, Oil Red O, Masson, Sirius Red, and α‐SMA antibody. Scale bar = 100 µm. The data were presented as the mean ± SD. Statistical differences were analyzed using an unpaired two‐tailed Student's t‐test or one‐way ANOVA followed by Tukey's post hoc test. ^*^ *p* < 0.05, ^**^ *p* < 0.01, ^***^ *p* < 0.001.

As a central regulator of cell growth, survival, and metabolism, the mTOR complex 1 (mTORC1) has been reported to enhance the translation of proteins that inhibit ferroptosis by promoting 4EBP1 phosphorylation [31, 32]. To determine whether PA suppresses hepatocyte ferroptosis through the mTORC1/4EBP1 pathway, we examined the phosphorylation levels of mTOR and 4EBP1 in mouse livers using WB analysis. As shown in Figure 6a, the MASLD group exhibited reduced phosphorylation of mTOR and 4EBP1 (p‑mTOR and p‑4EBP1) compared to the control group. In contrast, administration of either *B. uniformis* or PA increased p‐mTOR and p‐4EBP1 levels. Similarly, in THLE‐2 cells, RSL3 exposure inhibited mTOR and 4EBP1 phosphorylation, whereas both PA and Fer‐1 supplementation restored p‐mTOR and p‐4EBP1 expression (Figure 6b). To further validate whether mTORC1 activation is involved in PA‐mediated resistance to ferroptosis, we knocked down mTOR in THLE‐2 cells using siRNA (Figure S8). mTOR knockdown indeed sensitized THLE‐2 cells to RSL3‐induced ferroptosis, even in the presence of PA, leading to reduced GPX4 expression and increased Fe^2+^ accumulation, ROS levels, lipid peroxidation, and cell death (Figure 6c–i and Figure S9). Consistently, pharmacological inhibition of mTOR with the catalytic inhibitor Torin‐1 also counteracted the protective effects of PA against RSL3‐induced ferroptosis in THLE‐2 cells (Figures S10 and S11).

In alignment with these in vitro observations, co‑administration of Torin‑1 partially abolished the therapeutic effects of PA in HFHCD/CCl_4_‐challenged mice (Figure 6j,k). Compared to PA treatment alone, the addition of Torin‑1 resulted in elevated liver index, liver injury markers (ALT and AST), and lipid disorder markers (TC and TG). Moreover, the histological improvements mediated by PA, including reductions in hepatic steatosis, hepatocyte ballooning, lobular inflammation, HSC activation, and perisinusoidal fibrosis, were partially abolished by Torin‑1 co‑administration (Figure 6k,l). Together, these findings indicated that PA alleviated MASLD progression by boosting hepatocyte resistance to ferroptotic stress in an mTOR‑dependent manner.

## 3 Discussion

Polymorphic microbes have been recognized as a hallmark of chronic liver diseases [13, 14, 15]. However, research to date has largely focused on the gut microbiota, leaving the liver‐resident microbiota poorly understood. To our knowledge, this study provided the first characterization of the hepatic microbial landscape in the context of MASLD progression. We identified a distinct hepatic microbial signature in both human patients with advanced MASLD and corresponding mouse models, validating a consistent and significant reduction in hepatic *B. uniformis* compared to healthy controls. Parallel analysis revealed a concomitant decrease in gut *B. uniformis*, suggesting that diminished gut‐liver translocation of this bacterium might contribute to the development of advanced MASLD. In HFHCD/CCl_4_‐challenged mouse models, intragastric administration of *B. uniformis* could restore its hepatic abundance and exhibit probiotic functions against MASLD progression, as evidenced by marked resolutions of hepatic steatosis, hepatocyte ballooning, lobular inflammation, HSC activation, and perisinusoidal fibrosis. However, as the long‐term effects of *B. uniformis* administration on the overall microbial communities are not evaluated, whether the therapeutic benefits primarily result from the single strain itself or from a broader remodeling of the microbial communities remains unclear. Addressing this question will require further research, particularly prospective longitudinal profiling of microbial dynamics.

The past decade has witnessed a surge in descriptive profiling of microbial communities within internal organs once considered sterile [33, 34, 35]. These tissue‐resident microbes are now recognized not only for their role in maintaining physiological homeostasis, but also for their involvement in various diseases. In a seminal study, Cai et al. observed distinct microbial communities in malignant versus normal breast tissues, noting significant enrichment of *Staphylococcus*, *Streptococcus*, and *Lactobacillus* genera in breast tumors [33]. Strikingly, the investigators demonstrated that these intratumoral microbes could help protect cancer cells from fluid shear stress during circulation, thereby promoting metastasis. Similarly, pancreatic tissues have been shown to harbor diverse bacterial populations. McAllister et al. reported clear differences in tissue‐resident microbiota between long‐term and short‐term survivors of pancreatic ductal adenocarcinoma [34]. They identified a microbial signature including *Saccharopolyspora*, *Pseudoxanthomonas*, and *Streptomyces*, which was associated with improved survival, likely through enhancement of anti‐tumor immunity. Therefore, it is now believed that bacteria residing inside internal organs are not merely incidental bystanders. Instead, they appear to function as integral components of the tissue microenvironment, actively regulating host mechanisms to influence disease outcomes.

Another important issue is the origin of liver‐resident microbiota. While available researches confirm the presence of microbial communities in liver tissues, their sources remain poorly defined. In recent years, the bidirectional crosstalk and functional interdependence within the gut‐liver axis have become well‐established, with evidence suggesting that bacteria can translocate from the intestinal tract to the liver via the portal circulation [36, 37]. Supporting this, Yu et al. demonstrated that fecal microbiota transplantation from patients with HCC into mice enhanced gelatinase activity, thereby compromising intestinal epithelial integrity [37]. The intestinal barrier dysfunction facilitated colonization of the liver by viable, culturable gut microbes, which in turn upregulated oncogenic signaling pathways and promoted cancer cell proliferation. Parallel findings in cancerous pancreas suggested a similar gut‐derived origin for intrapancreatic bacteria, with gram‐negative *Proteobacteria* translocating directly from the duodenum into the pancreas via the pancreatic duct [38]. Such enhanced bacterial translocation might induce local dysbiosis and foster a tolerogenic immune microenvironment conducive to tumor growth. In this study, we detected common gut commensals, from the phylum *Bacteroidota* down to the species *B. uniformis*, in liver tissues. This observation provided evidence that bacterial translocation from the intestinal tract contributed to the establishment of liver‐resident microbiota. Additionally, the abundance of *B. uniformis* decreased synchronously in both liver and fecal samples during MASLD progression, suggesting that gut dysbiosis could shape hepatic microbial community via the gut‐liver axis. However, the promoter and ribosome binding site requirements in *B. uniformis* differ markedly from those of standard *E. coli* expression systems, and the prohibitive cost and long turnaround time of custom services ultimately preclude us from generating a fluorescently labeled strain for in vivo tracking. As a result, the evidence presented here remains indirect. Future studies employing advanced labeling techniques and in vivo imaging systems will be needed to obtain direct evidence that further substantiates these findings.

Our study advances the current understanding of *B. uniformis*. As one of the most abundant and prevalent species in gut microbiota, *B. uniformis* possesses a distinct ability to transform diverse polysaccharides into metabolites that regulate host energy balance, confer colonization resistance against intestinal pathogens, and maintain gut homeostasis [25, 26, 27]. Previous studies support a probiotic role for gut *B. uniformis*, as it is consistently depleted in various diseases, including inflammatory bowel disease, hypertriglyceridemic pancreatitis, allergic asthma, and autism spectrum disorder [39, 40, 41]. Bai et al. recently reported that *B. uniformis* was the most significantly reduced gut microbial species in mice with high‐fat diet‐induced hepatic steatosis [42]. They further demonstrated that *B. uniformis* supplementation improved liver function and lipid metabolism through the production of hexadecanedioic acid. However, the specific contribution of *B. uniformis* within liver‐resident microbiota has not been established. During MASLD progression, we observed a strong correlation between hepatic levels of *B. uniformis* and its metabolite PA. Notably, PA supplementation alone was sufficient to alleviate symptoms of advanced MASLD, supporting the idea that the therapeutic benefits were primarily mediated by the ability of hepatic *B. uniformis* to produce PA. However, we regret that intestinal barrier function, such as tight junction proteins and endotoxin levels, is not measured. Our study primarily focused on liver tissues and did not clarify whether *B. uniformis* improved gut barrier integrity to reduce pathogenic bacterial translocation, which could also explain the therapeutic effects of this bacterium. This remains an open question for future research.

Recent single‑cell RNA‑sequencing analyses indicate that hepatocytes exhibit a pronounced Fe^2+^ accumulation signature during MASLD progression, implying substantial ferroptotic stress in these cells [11, 43]. Ferroptotic hepatocytes lose regenerative capacity and adopt a ferroptosis‑associated secretory phenotype, which promotes lobular inflammation and perisinusoidal fibrosis via the secretion of proinflammatory and profibrogenic cytokines [7, 8, 9]. Given that both *B. uniformis* and its metabolite PA markedly attenuated lobular inflammation and perisinusoidal fibrosis in mouse models, we examined whether they reduced the liver burden of ferroptotic stress. Indeed, treatment with either *B. uniformis* or PA significantly reduced ferroptosis‑related markers in MASLD livers, even under continued HFHCD/CCl_4_ challenge. Importantly, in vitro experiments using THLE‑2 hepatocytes showed that PA substantially inhibited Fe^2+^ accumulation, lipid peroxidation, and cell death, mirroring the protective effects of ferroptosis inhibitor Fer‑1. No doubt, these findings demonstrate that the probiotic roles of hepatic *B. uniformis* against MASLD progression are mediated through the suppression of hepatocyte ferroptosis, thereby abrogating the proinflammatory and profibrogenic effects of ferroptosis‑associated secretory phenotype.

A key signaling hub integrating diverse metabolic and environmental cues to regulate protein synthesis is mTOR [44]. Previous studies have shown that ferroptotic cells exhibit sustained impairment of mTORC1 signaling, and that mTORC1 inhibition induces ferroptosis in various disease models. Upon activation, mTORC1 phosphorylates 4EBP1, releasing it from eIF4E and permitting 5′‐cap‐dependent translation initiation, which promotes protein synthesis and confers ferroptosis resistance [31, 32]. In this study, we observed that restoration of GPX4, a glutathione peroxidase that utilizes reduced GSH to detoxify lipid peroxides, contributed to the suppression of hepatocyte ferroptosis mediated by *B. uniformis* and PA. These findings prompted us to explore whether mTORC1 activation underlaid the therapeutic benefits of *B. uniformis* and PA against MASLD progression. Consistent with this hypothesis, our results showed that both *B. uniformis* and PA treatments significantly upregulated the mTORC1/4EBP1 pathway. Notably, mTOR inhibition via siRNA or the pharmacological inhibitor Torin‐1 reduced GPX4 expression in THLE‐2 hepatocytes and abrogated the anti‐ferroptotic effects of PA. Moreover, in mouse models, the ability of PA to alleviate the symptoms of advanced MASLD was abolished when blocking the activation of mTOR with Torin‐1. Therefore, the gut‐liver translocation of *B. uniformis*, its viability to produce PA, and the subsequent local activation of hepatic mTOR signaling are all critical for the probiotic function of this bacterium. The present study did not determine whether PA activated mTOR through direct binding or indirect regulation, leaving the upstream mechanisms incompletely understood. Potential mediators include the PI3K/AKT pathway, Rag GTPases, AMPK signaling, and HDAC inhibition. However, given the complexity and crosstalk among these pathways, the specific node(s) involved remain to be identified. In future work, we aim to systematically dissect these candidates using pharmacological inhibitors, siRNA‐mediated knockdown, and molecular interaction assays to definitively elucidate how PA activates mTOR.

Collectively, this study indicates that the liver‐resident microbiota plays an indispensable role in determining MASLD outcomes. A deeper understanding and characterization of this microbial community may inform the development of novel microbe‐targeted therapeutic strategies. Nevertheless, the small sample size and the lack of stratification by MASLD or fibrosis stage compromise the clinical representativeness of our findings, which should therefore be viewed as preliminary. Future multicenter, large‐sample studies using standardized protocols are warranted to perform stage‐specific stratification and validate our observations.

## 4 Experimental Section

The method details are available in Supplementary Materials.

## Author Contributions

H.L. performed data curation, formal analysis, investigation, validation, and wrote the original draft; Z.F. performed data curation, formal analysis, investigation, and wrote the original draft; Z.L. performed data curation, formal analysis, investigation, and validation; L.Q. performed supervision, validation, and edited the final manuscript; X.Z. performed conceptualization, methodology, supervision, validation, and edited the final manuscript.

## Funding

This research was supported by the National Natural Science Foundation of China (82000485), the Suzhou Gusu Health Talent Program (GSWS2021006), the Clinical Medicine Summit Program of the Suzhou Medical College of Soochow University (MA12201223), and the Boxi Talent Casting Program of the First Affiliated Hospital of Soochow University.

## Conflicts of Interest

The authors declare no conflicts of interest.

## Supporting information




**Supporting File 1**: advs76865‐sup‐0001‐SuppMat.docx.

## Data Availability

The data that support the findings of this study are available from the corresponding author upon reasonable request.
