# Supplementary material for: Gut‐Liver Translocation of Bacteroides Uniformis Alleviates Advanced Metabolic Dysfunction‐Associated Steatotic Liver Disease by Suppressing Hepatocyte Ferroptosis via Propionic Acid Secretion
Source: Adv Sci (Weinh). 2026 Jul 27;13(59):e76865. doi: 10.1002/advs.76865 (PMC13403911; doi:10.1002/advs.76865)
Supplement: Supplementary file 1 [file ADVS-13-e76865-s001.docx]

**Supplementary Materials**

**Gut-Liver Translocation of *Bacteroides uniformis* Alleviates Advanced Metabolic Dysfunction-Associated Steatotic Liver Disease by Suppressing Hepatocyte Ferroptosis via Propionic Acid Secretion**

Haiyang Liu^1^, Zun Fan^1^, Zhen Liu^1^, Lei Qin^1*^, and Xin Zhao^1*^

^1^Department of General Surgery, the First Affiliated Hospital of Soochow University, Suzhou, China.

^*^**Correspondence**:

Lei Qin (doctorqinlei@163.com), Xin Zhao (zx15152881615@163.com)

**1 Supplementary Methods**

**1.1 Human Studies**

This study enrolled patients who underwent liver resection at the Department of General Surgery, the First Affiliated Hospital of Soochow University from January 2024 to January 2025. A total of 30 participants were included, consisting of 15 patients with advanced MASLD (MASLD group) and 15 healthy controls (control group). The diagnosis of advanced MASLD was established according to the globally standardized consensus criteria, which require the coexistence of hepatic steatosis, significant fibrosis, and at least one cardiometabolic risk factor. Inclusion criteria were as follows: (1) age between 18 and 75 years; (2) evidence of hepatic steatosis on abdominal ultrasound; (3) confirmation of stage ≥ 2 fibrosis via imaging or histological examination; (4) presence of at least one cardiometabolic risk factor, including body mass index (BMI) ≥ 24 kg/m^2^, type 2 diabetes mellitus (T2DM), hypertension, or hyperlipidemia. Exclusion criteria included: (1) coexistence of other hepatic disorders such as viral hepatitis, alcoholic liver disease, drug‑induced liver injury, or autoimmune liver disease; (2) recent use (within 3 months prior to surgery) of medications that could potentially affect study outcomes, such as antibiotics, immunosuppressants, chemotherapeutic agents, or proton pump inhibitors; (3) severe concomitant cardiovascular or cerebrovascular diseases.

All human liver tissues were collected under sterile conditions using surgical scalpels. Samples from the control group were obtained from normal areas adjacent to hepatic hemangiomas or focal nodular hyperplasia. Samples from the MASLD group were harvested from non-tumorous regions surrounding HCC that had developed in the setting of advanced MASLD. Immediately after collection, one portion of each sample was snap‑frozen in liquid nitrogen for 16S rRNA sequencing, while the remaining portion was fixed in formalin and paraffin‑embedded for histological analysis. This study protocol was approved by the Institutional Review Board of the First Affiliated Hospital of Soochow University (Approval Number: 2026329). All procedures were conducted in strict accordance with the ethical guidelines outlined in the Helsinki Declaration. Written informed consent was obtained from each participant prior to enrollment.

**1.2 Animal Experiments**

Male C57BL/6J mice, aged 6-8 weeks, were obtained from the Experimental Animal Center of Soochow University. All animal experiments were conducted in accordance with protocols approved by the Animal Ethics Committee of Soochow University (Approval Number: 202502A0036). After one week of acclimatization, the mice were divided into six groups (n = 6 for each group): control, MASLD, *B. uniformis*, non-viable *B. uniformis*, PA, and Torin‑1 groups. The control group received a normal chow diet along with weekly intraperitoneal injections of olive oil for 12 weeks. The other five groups were fed a HFHCD (42% kcal from fat, 1.25% cholesterol) and administered weekly intraperitoneal injections of CCl_4_ (diluted in olive oil, 2 µL/g body weight) for 12 weeks to induce advanced MASLD.

Beginning in week 9, interventions were administered for 4 weeks. Mice in the *B. uniformis* and non-viable *B. uniformis* groups received oral gavage of 200 µL suspension containing live or pasteurized *B. uniformis* (2×10^8^ CFU/mL in saline), respectively, twice per week. The PA and Torin-1 groups both received drinking water supplemented with 200 mM PA (HY-B1773A, MedChemExpress, USA), while the Torin-1 group additionally received daily intraperitoneal injections of 20 mg/kg Torin-1 (HY-13003, MedChemExpress, USA). Three days after the final intervention, fecal samples were collected. Following an overnight fast, mice were anesthetized and euthanized. Serum and liver samples were harvested for subsequent analysis.

**1.3 16S rRNA Sequencing**

Stringent contamination control is essential when working with low microbial biomass tissues such as the liver. (1) Negative control setup: During liver tissue collection from each patient, a cryovial from the same batch, containing sterile saline, was opened on the operating table alongside the tissue sample, and exposed to the same environmental conditions for the same duration. The cryovial was then immediately capped, transported together with the tissue samples in liquid nitrogen, and served as a sampling blank control. This control was designed to evaluate background contamination that could potentially originate from the operating table environment, the sampling containers, or the transport process. In addition, for 16S rRNA sequencing, DNA extraction controls and PCR amplification controls were included in every batch. (2) Contamination control strategy: To capture potential contamination introduced throughout the entire sample handling workflow, all negative controls (including sampling blank controls, DNA extraction controls, and PCR amplification controls) were processed in parallel with the actual tissue samples during DNA extraction, PCR amplification, library preparation, and sequencing. Contamination was generally considered absent when negative control samples showed no visible band on an agarose gel. Negative control data that passed quality control were included in subsequent background bacterial identification, whereas those that failed quality control were excluded from the contamination assessment. Quality control of the raw data involved filtering out samples with a total read count below 1,000 and species with a relative abundance below 1×10^−4^. To account for the most prevalent general contaminants, any taxa present in more than 30% of negative controls was completely removed.

Microbial DNA was extracted from liver and fecal samples using the FastPure Fecal DNA Extraction Kit (Major Bio, China). DNA integrity was evaluated by 1% agarose gel electrophoresis, and concentration and purity were determined using a NanoDrop 2000 spectrophotometer (Thermo Scientific, USA). Sample-specific amplification strategies were applied. For liver samples, multiplex PCR with barcoded primers targeting the hypervariable regions V2, V3, V5, V6, and V8 of the 16S rRNA gene was performed, whereas fecal samples were amplified for the V3-V4 variable regions (Table S3). Purified PCR products were used for library preparation with the NEXTFLEX® Rapid DNA-Seq Kit (Bioo Scientific, USA), followed by sequencing on an Illumina NextSeq 2000 platform (Illumina, USA). Microbial richness was analyzed by comparing α-diversity using the Wilcoxon rank‑sum test based on the Chao1, Shannon, and Simpson indices. β‑Diversity was evaluated by PCoA using Bray-Curtis distances, with statistical significance assessed via PERMANOVA. Differential abundance at the genus level was identified using the Wilcoxon rank‑sum test with p values adjusted with Bonferroni correction, and an adjusted p value (p_adj) < 0.05 was considered statistically significant. Additionally, LEfSe was employed to identify bacterial taxa showing significant abundance differences from phylum to species level between groups.

**1.4 FISH Assay**

Formalin-fixed paraffin-embedded liver sections were deparaffinized, rehydrated, and treated with 10 mg/mL proteinase K for 10 min at room temperature. Overnight hybridization was carried out at 37 ℃ using a universal bacterial 16S rRNA probe (EUB338, labeled with Cy5) and a *B. uniformis*-specific probe (labeled with Texas Red), each at a final concentration of 10 ng/μL (Table S4). After three washes with wash buffer, the sections were mounted with an antifade medium containing DAPI, and images were acquired using a fluorescent microscope.

**1.5 DNA Extraction and PCR Quantification**

Genomic DNA from liver and fecal samples was extracted using the SteadyPure Bacteria Genomic DNA Extraction Kit (AG21008, Accurate Biotechnology, China) according to the manufacturer’s instructions. DNA concentration and purity were measured using a NanoDrop 2000 spectrophotometer. Subsequently, qPCR was performed using Hieff UNICON® Universal Blue qPCR SYBR Green Master Mix (11184ES08, Yeasen, China) on a LightCycler 480 real-time PCR system (Roche Diagnostics, Switzerland). Primers used for *B. uniformis* amplification were listed in Table S5.

**1.6 SCFA Targeted Metabolomics Analysis**

Quantification of SCFAs in liver samples and culture supernatants was conducted by liquid chromatography-tandem mass spectrometry (LC-MS/MS). Approximately 20 mg of frozen liver samples or 50 μL of culture supernatants was extracted with 500 μL of methanol-water (4:1, v/v). Liver samples were homogenized using a cryogenic grinder at -10 ℃ (50 Hz, 6 min). The homogenates were subsequently sonicated at 5 ℃ for 30 min, allowed to equilibrate at room temperature for 30 min, and centrifuged at 13,000 ×g for 15 min at 4 ℃. An aliquot of 20 μL supernatants was derivatized by adding 20 μL of 200 mM 3-nitrophenylhydrazine hydrochloride and 20 μL of 120 mM 1-ethyl-3-(3-dimethylaminopropyl) carbodiimide hydrochloride containing 6% pyridine, followed by incubation at 40 ℃ for 30 min. The derivatized mixture was then diluted to 1 mL with 50% aqueous acetonitrile before LC-MS/MS analysis. Separation was achieved on a reversed-phase column, and detection was performed in negative electrospray ionization mode with multiple reaction monitoring. Quantification was based on external calibration curves, with deuterated butyrate‑d8 as an internal standard.

**1.7 Bacterial Culture and Suspension Preparation**

*B. uniformis* (strain BNCC139204) was obtained from the BeNa Culture Collection (China). Anaerobic conditions were established using Mitsubishi anaerobic gas production bags and anaerobic culture bags. The strain was inoculated onto Columbia blood agar plates and incubated anaerobically at 37 ℃ for 24 h. Bacterial cells were harvested, washed, and resuspended in sterile, anaerobic phosphate-buffered saline (PBS). The suspension was adjusted to a concentration of 2×10^8^ CFU/mL and stored at 4 ℃ until further use. Non-viable *B. uniformis* was prepared by pasteurization at 65 ℃ for 30 min.

**1.8 Histological and IHC Staining**

Liver tissue samples were fixed in formalin, embedded in paraffin, and sectioned at a thickness of 4 μm. According to routine protocols, H&E, Masson, and Sirius Red staining were performed to evaluate hepatocyte ballooning, lobular inflammation, and perisinusoidal fibrosis. For lipid accumulation analysis, frozen liver tissues were sectioned at a thickness of 10 μm and stained with Oil Red O following established procedures. TUNEL staining was performed with a One Step TUNEL Apoptosis Assay Kit (KGA1400, KeyGEN BioTECH, China) according to the manufacturer’s instructions. For IHC staining, deparaffinized and rehydrated sections were incubated with primary antibodies against α-SMA (1:100; 41550, Signalway Antibody, USA), 4-HNE (1:500; GB150073, Servicebio, China), and CD11b (1:500; GB15058, Servicebio, China). Following HRP-conjugated secondary antibody incubation, DAB substrate solution was added for visualization. Quantitative analysis was performed using Image J (V1.8.0, NIH, USA).

**1.9 Biochemical Detection**

The serum levels of ALT (A009-2-1), AST (C010-2-1), TC (A111-1-1), and TG (A110-1-1) in mice were measured using commercial kits provided by NJJCBIO (China). All detection procedures were carried out strictly in accordance with the instructions provided with the reagent kits. The concentration of Fe^2+^ in mouse liver tissues was measured using the Tissue Iron Assay Kit (A039-2-1) from NJJCBIO (China). Briefly, the samples were homogenized, centrifuged at 12,000 ×g for 5 min at 4 ℃, and the resulting supernatant was collected for analysis.

**1.10 ELISA Detection**

The levels of TNF-α (CSB-E04741m), IL-6 (CSB-E04639m), and TGF-β (CSB-E09785m) in mouse liver tissues were determined using ELISA kits (CUSABIO, China). The detection was conducted following the instructions of manufacturer’s protocols. The absorbance was measured using a microplate reader (Epoch 2, BioTek Instruments, USA) at 450 nm.

**1.11 GSH/GSSG Assay**

The levels of reduced GSH and GSSG in mouse liver samples and THLE-2 cells were measured using a commercial GSH/GSSG Assay Kit (S0053, Beyotime, China) according to the manufacturer’s instructions. Briefly, liver tissues and cultured cells were collected, washed with PBS, and subsequently homogenized or lysed. The homogenates were centrifuged at 10,000 ×g for 5 min at 4 ℃, and the resulting supernatants were used for determination of the GSH/GSSG ratio.

**1.12 Cell Culture and Treatment**

The human immortalized hepatocyte line THLE-2 was obtained from Procell Biotechnology (CL-0833, China). Cells were cultured in THLE-2 complete medium (CM-0833, Procell Biotechnology, China) at 37 ℃ in a humidified incubator containing 5% CO_2_ and routinely passaged at 70-80% confluence. Ferroptosis was induced by treating THLE-2 cells with 1 μM RSL3 (M9060, AbMole, USA) for 24 h. To investigate the protective effects of PA against ferroptosis, cells exposed to RSL3 were co-treated for 24 h with 5 mM PA, 10 μM Fer-1 (M2698, AbMole, USA), or a combination of both agents.

**1.13 CCK-8 Assay**

Cell viability was assessed using a CCK-8 kit (M4839, AbMole, USA). THLE-2 cells were seeded in 96-well plates at a density of 1×10^4^ cells per well and treated as indicated for 24 h. Following incubation with CCK-8 reagent for 1 h at 37 ℃, the absorbance at 450 nm was measured using a microplate reader.

**1.14 Flow Cytometry**

THLE-2 cells were seeded into 6-well plates at a density of 2×10^5^ cells per well. After indicated treatments, intracellular ROS levels were evaluated using DCFH-DA (D6470, Solarbio, China), lipid peroxidation was assessed with C11-BODIPY 581/591 (B3930, Solarbio, China), and apoptosis rates were determined by Annexin V-FITC/PI staining (M55205, AbMole, USA), in accordance with the manufacturers’ protocols. Fluorescent signals were acquired on a BD FACSCelesta flow cytometer (BD Biosciences, USA) and analyzed using FlowJo (V10.8.1, BD Biosciences, USA).

**1.15 Intracellular Fe^2+^ Detection**

Intracellular Fe^2+^ levels were measured using the FerroOrange fluorescent probe (M42835, AbMole, USA) according to the manufacturer’s instructions. Briefly, cells were washed with PBS, stained with 1 μM FerroOrange for 30 min at 37 ℃, and subsequently imaged using a fluorescent microscope (Celldiscoverer 7, Carl Zeiss AG, Germany).

**1.16 TEM Observation**

THLE-2 cells were fixed in 2.5% glutaraldehyde, postfixed in 1% osmium tetroxide, dehydrated through a graded ethanol series. Subsequently, the samples were embedded in epoxy resin and sectioned at a thickness of 70 nm. The sections were stained with uranyl acetate and lead citrate and examined using a TEM (Tecnai G2 F20, FEI, USA).

**1.17 SiRNA Transfection**

Human mTOR pre-designed siRNA (HY-RS08810, MedChemExpress, USA) was used for gene silencing. The siRNA sequence was provided in Table S6. THLE-2 cells were seeded in 6-well plates at a density of 3×10^5^ cells per well and transiently transfected with 50 nM siRNA using Lipofectamine 3000 (Thermo Scientific, USA) according to the manufacturer’s instructions. After transfection, cells were cultured for an additional 48 h for subsequent experiments.

**1.18 WB Analysis**

Total protein was extracted from tissues and cells using lysis buffer, followed by centrifugation at 12,000 ×g for 10 min at 4 ℃. Protein concentration was determined using a BCA Assay Kit (KIT-BCA01, Sino Biological Inc., China). Equal amounts of protein were denatured, separated by SDS-PAGE, and transferred onto polyvinylidene fluoride membranes. Membranes were then blocked with 5% non-fat milk and incubated overnight at 4 ℃ with primary antibodies, followed by incubation with HRP-conjugated secondary antibody for 1 h at room temperature. Protein bands were visualized using enhanced chemiluminescence reagents. Primary antibodies included: xCT (1:1000; ab307601, Abcam, USA), GPX4 (1:1000; ab125066, Abcam, USA), mTOR (1:2000; YM8208, Immunoway, USA), p-mTOR (Ser2448; 1:1000; YP0176, Immunoway, USA), 4EBP1 (1:2000; YM8369, Immunoway, USA), p-4EBP1 (Ser65; 1:1000; YP0618, Immunoway, USA), and β-actin (1:2000; 100166-MM10, Sino Biological Inc., China).

**1.19 Statistical Analysis**

The data were expressed as mean ± standard deviation (SD). Two-group comparisons were performed using an unpaired two-tailed Student’s t test. Differences between multiple groups were compared using a one-way analysis of variance (ANOVA) followed by Tukey’s post hoc test. All analyses were performed using GraphPad Prism 7.0 (GraphPad Software, USA). The p value of < 0.05 was regarded as a significance threshold.

**2 Supplementary Figures**

**Figure S1.** LEfSe analysis identifying differentially abundant microbial species in human liver samples between the control and MASLD groups (n = 15).

**Figure S2.** LEfSe-derived cladogram identifying differentially abundant taxa across all taxonomic levels in human fecal samples between the control and MASLD groups (n = 15).

**Figure S3.** Effects of non-viable *B. uniformis* on MASLD progression. (a) Live/dead bacterial staining with DMAO (green) and PI (red) to evaluate the viability of *B. uniformis* after pasteurization. Scale bar = 10 μm. (b) Schematic of experimental design, with purple arrows indicating oral gavage of non-viable *B. uniformis* (n = 6). (c) Representative images of mouse liver samples after staining with H&E, Oil Red O, Masson, Sirius Red, and α-SMA antibody. Scale bar = 100 μm. (d) Quantitative analysis of liver index, serum ALT, AST, TC, and TG levels, as well as positive areas of Oil Red O, Masson, Sirius Red, and α-SMA staining between the *B. uniformis* and non-viable *B. uniformis* groups (n = 6). The data were presented as the mean ± SD. Statistical differences were analyzed using an unpaired two-tailed Student’s t test. *p < 0.05, **p < 0.01, ***p < 0.001.

**Figure S4**. Bubble plot highlighting the differentially expressed SCFAs in culture medium from *B. uniformis* before and after pasteurization.

**Figure S5.** (a) CCK-8 assay to evaluate the cell viability in THLE-2 hepatocytes treated with increasing concentrations of PA. (b) CCK-8 assay to evaluate the cell viability in RSL3-induced THLE-2 hepatocytes following treatment with increasing concentrations of PA. The data were presented as the mean ± SD. Statistical differences were analyzed using a one-way ANOVA followed by Tukey’s post hoc test. **p < 0.01, ***p < 0.001, NS: not significant.

**Figure S6**. (a) ELISA to measure the protein levels of TNF-α, IL-6, and TGF-β in mouse liver samples from the control, MASLD, *B. uniformis*, and PA groups (n = 6). (b) Representative images of CD11b staining in mouse liver samples from the four groups. Scale bar = 100 μm. (c) Quantitative analysis of CD11b positive areas (n = 6). The data were presented as the mean ± SD. Statistical differences were analyzed using a one-way ANOVA followed by Tukey’s post hoc test. ***p < 0.001.

**Figure S7.** After being stained with FerroOrange (Fe^2+^, red) and Hoechst 33342 (cell nucleus, blue), fluorescent images of THLE-2 hepatocytes were captured from the control, PA, RSL3, RSL3 + PA, RSL3 + Fer-1, and RSL3 + PA + Fer-1 groups. Scale bar = 20 μm.

**Figure S8.** WB analysis showing the silencing efficiency of three independent siRNAs targeting mTOR (simTOR-1-3) in THLE-2 hepatocytes.

**Figure S9.** PA suppressed hepatocyte ferroptosis by targeting mTOR. (a) Annexin V/PI staining to evaluate the cell apoptosis in RSL3-induced THLE-2 hepatocytes treated with siCtr, PA + siCtr, simTOR, or PA + simTOR. (b) FerroOrange fluorescence to evaluate the Fe^2+^ accumulation in RSL3-induced THLE-2 hepatocytes subjected to different treatments. Scale bar = 20 μm. (c) DCFH-DA staining to evaluate the ROS levels in RSL3-induced THLE-2 hepatocytes subjected to different treatments. (d) C11-BODIPY staining to evaluate the lipid peroxidation in RSL3-induced THLE-2 hepatocytes subjected to different treatments.

**Figure S10.** Torin-1 abolished the protective effects of PA against RSL3-induced ferroptosis in THLE-2 hepatocytes. (a) CCK-8 assay to evaluate the cell viability in RSL3-induced THLE-2 hepatocytes treated with PBS, PA, Torin-1, or PA + Torin-1 (n = 3). (b) Annexin V/PI staining to evaluate the cell apoptosis in RSL3-induced THLE-2 hepatocytes subjected to different treatments. (c) Quantitative analysis of cell apoptosis in RSL3-induced THLE-2 hepatocytes subjected to different treatments (n = 3). (d) FerroOrange fluorescence to evaluate the Fe^2+^ accumulation in RSL3-induced THLE-2 hepatocytes subjected to different treatments (n = 3). Scale bar = 20 μm. The data were presented as the mean ± SD. Statistical differences were analyzed using a one-way ANOVA followed by Tukey’s post hoc test. **p < 0.01, ***p < 0.001.

**Figure S11.** Torin-1 abolished the protective effects of PA against RSL3-induced ferroptosis in THLE-2 hepatocytes. (a) DCFH-DA staining to evaluate the ROS levels in RSL3-induced THLE-2 hepatocytes treated with PBS, PA, Torin-1, or PA + Torin-1 (n = 3). (b) C11-BODIPY staining to evaluate the lipid peroxidation in RSL3-induced THLE-2 hepatocytes subjected to different treatments (n = 3). (c) WB analysis of mTOR, p‑mTOR, 4EBP1, and p‑4EBP1 in RSL3-induced THLE-2 hepatocytes subjected to different treatments (n = 3). (d) WB analysis of xCT and GPX4 in RSL3-induced THLE-2 hepatocytes subjected to different treatments (n = 3). The data were presented as the mean ± SD. Statistical differences were analyzed using a one-way ANOVA followed by Tukey’s post hoc test. *p < 0.05, **p < 0.01.

**3 Supplementary Tables**

**Table S1.** Clinical and demographic characteristics of included patients with advanced MASLD and healthy controls.

|  | Overall | MASLD | Control |
| --- | --- | --- | --- |
| Characteristic | n = 30 | n = 15 | n = 15 |
| Age (years)  median (IQR) | 55 (42-70) | 55 (45-71) | 52 (38-66) |
| Sex, female | 16 (53.3) | 7 (46.7) | 9 (60.0) |
| Smoking status |  |  |  |
| Never | 27 (90) | 13 (86.7) | 14 (93.3) |
| Previous | 0 (0) | 0 (0) | 0 (0) |
| Current | 3 (10) | 2 (13.3) | 1 (6.7) |
| Medication for T2DM | 12 (40) | 9 (60) | 3 (20) |
| Medication for  hypertension | 17 (56.7) | 13 (86.7) | 4 (26.7) |
| BMI (kg/m^2^)  median (IQR) | 24.2 (21.8-25.9) | 25.2 (23.4-26.6) | 23.3 (21.2-25.2) |

Data were presented as the n (%) unless otherwise specified. IQR: interquartile range, BMI: body mass index, T2DM: type 2 diabetes mellitus.

**Table S2.** Baseline characteristics of participants providing fecal samples from the metagenomic datasets of NCBI SRA/ENA accessions PRJNA373901 and PRJEB6070.

|  | Overall | MASLD | Control |
| --- | --- | --- | --- |
| Characteristic | n = 30 | n = 15 | n = 15 |
| Age (years)  median (IQR) | 54 (47-65) | 47 (43-57) | 59 (54-65) |
| Sex, female | 10 (33.3) | 5 (33.3) | 5 (33.3) |
| BMI (kg/m^2^)  median (IQR) | 30.9 (27.8-34.3) | 34.3 (28.3-37.9) | 30.2 (23.1-30.9) |

Data were presented as the n (%) unless otherwise specified. IQR: interquartile range, BMI: body mass index.

**Table S3.** 16S rRNA amplification primer sequences used in this study.

| Target  region | Forward  primer (5'-3') | Reverse  primer (5'-3') |
| --- | --- | --- |
| V2 | TGGCGAACGGGTGAGTAA | CCGTGTCTCAGTCCCARTG |
| V3 | ACTCCTACGGGAGGCAGC | GTATTACCGCGGCTGCTG |
| V5 | GTGTAGCGGTGRAATGCG | CCCGTCAATTCMTTTGAGTT |
| V6 | GGAGCATGTGGWTTAATTCGA | CGTTGCGGACTTAACCC |
| V8 | GGAGGAAGGTGGGGATGAC | AAGGCCCCGGAAACGTATT |
| V3-V4 | ACTCCTACGGGAGGCAGCAG | GGACTACHVGGGTWTCTAAT |

Degenerate bases follow IUPAC nomenclature (R = A/G, W = A/T, H = A/C/T, M = A/C, V = A/C/G).

**Table S4.** FISH probe sequences used in this study.

| Probe | Sequence (5'-3') | Dye |
| --- | --- | --- |
| EUB338 | GCTGCCTCCCGTAGGAGT | Cy5 |
| *B. uniformis* | GACATGTCTCCACATCATTCAGT | Texas Red |

**Table S5.** qPCR primer sequences used in this study.

| Target species | Forward primer (5'-3') | Reverse primer (5'-3') |
| --- | --- | --- |
| *B. uniformis* | TCTTCCGCATGGTAGAACTATTA | ACCGTGTCTCAGTTCCAATGTG |

**Table S6.** siRNA sequences targeting mTOR used in this study.

| Target gene | Sense (5'-3') | Antisense (5'-3') |
| --- | --- | --- |
| simTOR-1 | GCUGUGCUACACUACAAACAUTT | AUGUUUGUAGUGUAGCACAGCTT |
| simTOR-2 | CCAAGAUACCAUGAACCAUTT | AUGGUUCAUGGUAUCUUGGAG |
| simTOR-3 | CCAGCCAAUCAUUCGCAUUTT | AAUGCGAAUGAUUGGCUGGTT |

siRNA sequences were shown in RNA notation (U instead of T), with TT overhangs.
